# The role of long non-coding RNA HCP5 in ulcerative cutaneous tuberculosis therapy introduction

**DOI:** 10.3389/fcimb.2026.1824185

**Published:** 2026-06-11

**Authors:** Jiayan Qian, Jiayue Sun, Ye Liu, Qianqian Bu, Tingjun Cao, Quanyong Jin, Liangjie Fu, Yuling Wang, Xianyu Gong, Rui Deng, Zihui Huang

**Affiliations:** 1 Affiliated of Nanjing University of Chinese Medicine to Nanjing Integrated Traditional Chinese and Western Medicine Hospital, Jiangsu, Nanjing, China; 2 Jiangsu Second Chinese Medicine Hospital, Jiangsu, Nanjing, China; 3 Maqun Community Health Service Center, Jiangsu, Nanjing, China

**Keywords:** biomarker, ITGB2, LncRNA HCP5, therapeutic monitoring, ulcerative cutaneous tuberculosis

## Abstract

**Background:**

Ulcerative Cutaneous Tuberculosis (UCT) represents a diagnostically complex variant of extrapulmonary tuberculosis, characterized by the absence of standardized biomarkers for monitoring treatment efficacy.

**Objective:**

This study sought to explore the potential of lncRNA *HCP5* as a diagnostic and therapeutic biomarker for UCT.

**Methods:**

We conducted an integrated bioinformatics analysis on clinical samples from patients with UCT and Non-Tuberculous Cutaneous Ulcers to identify differentially expressed lncRNAs. Co-expression networks were developed to associate HCP5 with downstream mRNAs. Validation was performed using patient tissue samples and an *in vitro* model of BCG-infected macrophages, incorporating functional knockdown studies. Longitudinal profiling of UCT patients undergoing therapy was employed to evaluate the dynamics of the biomarker.

**Results:**

Our investigation identified 1,994 differentially expressed long non-coding RNAs (lncRNAs), with HCP5 being notably upregulated. Through co-expression analysis, HCP5 was linked to immune and inflammation pathways, and 12 correlated mRNAs were identified, among which ITGB2 was validated as a downstream target. The knockdown of HCP5 resulted in a significant reduction of ITGB2 expression in macrophages. Longitudinal analysis demonstrated a significant decline in both HCP5 and ITGB2 levels in patient tissues and plasma following two weeks of anti-tuberculosis treatment.

**Discussion:**

This study elucidates a novel HCP5/ITGB2 regulatory axis in the pathogenesis of UCT and highlights their dynamic responsiveness to therapeutic intervention. These findings address a critical clinical gap by providing objective biomarkers for the diagnosis and monitoring of treatment efficacy in cutaneous tuberculosis.

## 1 Introduction

Tuberculosis (TB) is a highly infectious disease with a significant global incidence, constituting a major public health challenge worldwide (Motta et al., 2024; Yao Y, et al., 2025; Li et al., 2026). It disproportionately impacts populations with low socioeconomic status and marginalized communities. It is estimated that over one-third of the global population is latently infected with *Mycobacterium tuberculosis*, the pathogen responsible for TB (McCaffrey et al., 2026). According to the World Health Organization’s “Global Tuberculosis Report 2025” (WHO, 2025), there were 10.7 million new TB cases and 1.23 million TB-related deaths worldwide in 2024. Furthermore, the extensive use of anti-TB drugs has facilitated the emergence of drug-resistant Mycobacterium tuberculosis strains. As a result, TB incidence remains persistently high, making it a prevalent and complex disease that poses a significant threat to global health (Schwalb et al., 2026).

Ulcerative cutaneous tuberculosis (UCT) represents a manifestation of extrapulmonary tuberculosis, predominantly induced by *Mycobacterium tuberculosis* (*M. tb*) through mechanisms such as direct inoculation, local tissue spread, or lymphatic/hematogenous dissemination, culminating in a chronic non-healing wound (Gramminger and Biedermann, 2025). The clinical presentation of UCT is frequently marked by atypical symptoms, diverse morphological characteristics, and recurrent pseudo-cicatrization, which pose substantial challenges for both diagnosis and treatment (Huang et al., 2022). The prevalence of UCT is notably elevated in regions burdened with high rates of tuberculosis, HIV infection, connective tissue diseases, and multidrug-resistant TB (MDR-TB) (Rathored et al., 2026), thereby presenting significant obstacles for TB control programs, public health systems, and regional economic development (Hailu et al., 2024; Mu et al., 2026).

Despite the existence of well-established treatment regimens for ulcerative cutaneous tuberculosis, the rate of surgical intervention remains high. This is primarily attributable to the challenges in clinical diagnosis, often resulting in delayed treatment and progression of the disease to advanced stages, thereby increasing the necessity for surgical intervention (Kaul et al., 2023). A critical factor contributing to this issue is the absence of sensitive and specific biomarkers for early diagnosis. Consequently, the early diagnosis and effective intervention are pivotal for enhancing therapeutic outcomes (Theobald et al., 2025; Keren et al., 2025).

Long non-coding RNAs (lncRNAs) have been identified as key regulators in the host immune response to *Mycobacterium tuberculosis* (M. *tb*). Research has shown that M. *tb* infection leads to the dysregulation of lncRNAs involved in immune regulatory pathways (Vijayaraghavan et al., 2024; Li et al., 2025; Gong et al., 2025). A growing body of evidence suggests that lncRNAs serve as essential coordinators of various biological processes, including immune responses and host-pathogen interactions. Despite this, the potential application of lncRNAs as biomarkers for diagnosis, monitoring disease progression, and assessing treatment response remains inadequately defined in both clinical and laboratory contexts (Yao S, et al., 2025; Ji et al., 2026; Vijayaraghavan et al., 2024).

LncRNAs can act as competitive endogenous RNAs (ceRNAs) within lncRNA-miRNA-mRNA networks, influencing target gene expression and thereby contributing to the pathogenesis and progression of immune-related diseases (Lan et al., 2025). Notably, the lncRNA HLA Complex P5 (HCP5) is a significant transcript located within the Major Histocompatibility Complex (MHC) Class I region, specifically positioned between the MHC Class I polypeptide-related sequence A (MICA) and B (MICB) genes.

It is predominantly expressed in immune cells, where it plays a role in both adaptive and innate immune responses, and demonstrates high and specific expression within lymphoid tissues (Kulski, 2019; Li et al., 2024). In our previous investigation comparing ulcerative cutaneous tuberculosis (UCT) to non-tuberculous cutaneous ulcer (NTCU) tissues, we identified a significantly elevated expression of the long non-coding RNA (lncRNA) HCP5 in UCT lesions. Additionally, research indicates that HCP5 expression is upregulated in THP-1 macrophages subsequent to Mycobacterium tuberculosis infection. A substantial body of evidence links HCP5 overexpression with the clinicopathological features and prognosis of various diseases (Li et al., 2024; Wang et al., 2022). This evidence underscores the potential of HCP5 as a biomarker for the diagnosis, monitoring of disease progression, and assessment of treatment efficacy in ulcerative cutaneous tuberculosis (Hu et al., 2021).

In this study, transcriptomic analysis was utilized to analyze tissue specimens from UCT and NCTU. The NCTU cohort primarily comprised pressure ulcers, non-healing surgical wounds, venous leg ulcers, and cutaneous soft-tissue infections. These conditions were selected as controls because, despite closely mimicking UCT in clinical presentation (both are chronic, non-healing wounds), they differ fundamentally in etiology. Furthermore, advanced NCTU lesions are often associated with localized immune deficiency and prolonged wound exposure, which heighten the risk of Mycobacterium tuberculosis superinfection and may delay appropriate treatment. By using NCTU as a comparator, we aimed to isolate UCT-specific molecular signatures distinct from general ulcer-associated inflammatory changes. Based on this design, we constructed an lncRNA-miRNA-mRNA regulatory network centered on HCP5 to identify key genes involved in UCT pathogenesis. Finally, we evaluated the translational potential of lncRNA HCP5 as a biomarker for the diagnosis, disease monitoring, and therapeutic assessment of UCT.

## 2 Materials and methods

The study protocol was approved by the Ethics Committee of Nanjing Hospital of Integrated Traditional Chinese and Western Medicine (Nanjing, China) on September 27, 2021 (Approval No: 2021037). All patients provided written informed consent for the use of their samples and data in this study.

### 2.1 Clinical sample collection

**Figure 1:**
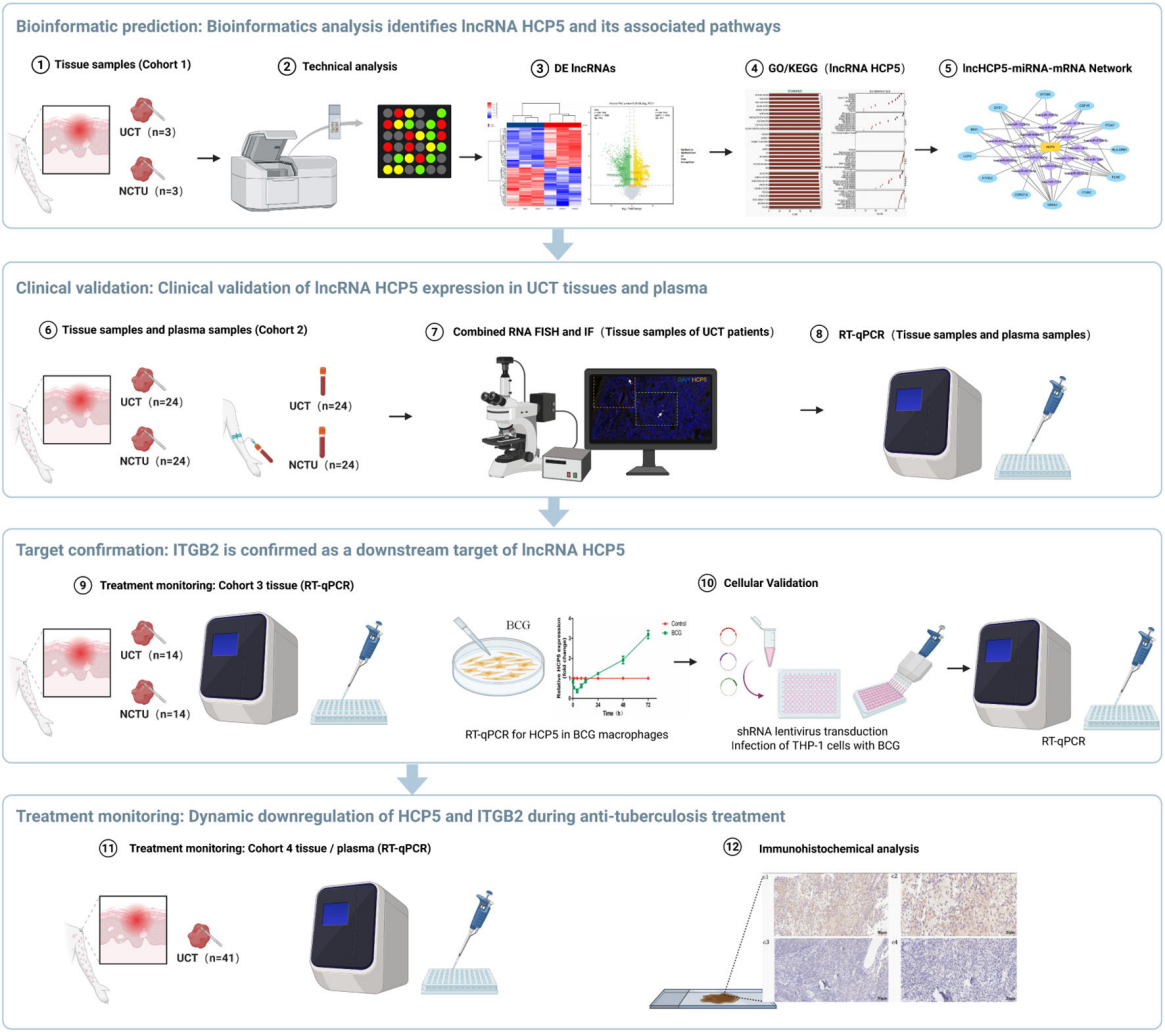
Schematic workflow of the study. UCT, ulcerative cutaneous tuberculosis; NCTU, non-tuberculous cutaneous ulcer; DE, differentially expressed; GO, Gene Ontology; KEGG, Kyoto Encyclopedia of Genes and Genomes; FISH, RNA *in situ* hybridization; IF, immunofluorescence; RT-qPCR, real-time reverse transcription-quantitative polymerase chain reaction; BCG, Bacillus Calmett-Guérin; IHC, immunohistochemistry.

Lesion tissue samples were initially collected from 3 patients diagnosed with UCT and 3 patients with NCTU at the Nanjing Hospital of Integrated Traditional Chinese and Western Medicine between 2019 and 2020. This initial cohort, referred to as Cohort 1, was utilized for transcriptomic analysis. Subsequently, a second cohort comprising 48 patients (24 with UCT and 24 with NCTU) was recruited from 2021 to 2022 (Cohort 2) to validate the previously identified elevated expression of lncRNA HCP5 in UCT tissues. Furthermore, a third cohort comprising 28 patients (14 with UCT and 14 with NCTU) was recruited from 2022 to 2023 (Cohort 3) to explore the downstream target genes associated with lncRNA HCP5. Additionally, from 2022 to 2023, a fourth distinct cohort of 41 patients with UCT (Cohort 4) was enrolled to assess biomarker dynamics during treatment. For Cohort 4, paired lesion tissue and plasma samples were collected before and after a standardized 2-week anti-tuberculosis treatment regimen to evaluate longitudinal changes in the expression of lncRNA HCP5 and ITGB2. The overall study workflow was illustrated in **Figure 1**.

All participants underwent a standardized diagnostic evaluation, which included clinical examination, basic laboratory tests, histopathological examination of lesion tissues, and chest computed tomography. The baseline demographic and clinical characteristics of the four cohorts are summarized in **Table 1**. Patients with UCT and NCTU were matched and exhibited no significant differences in baseline demographic or clinical characteristics, including age, gender, smoking status, alcohol consumption, or comorbidities. Notably, none of the patients in the UCT groups (Cohorts 1, 2, 3 and 4 at baseline) had received anti-tuberculosis treatment prior to their enrollment in the study. The exclusion criteria for all cohorts included: (1) active pulmonary or extrapulmonary tuberculosis, apart from the presenting UCT; (2) significant psychiatric illness; (3) known impaired immune function, such as symptomatic HIV infection, hematological malignancy, or systemic malignancy; and (4) current use of immunosuppressive therapy, including corticosteroids, alkylating agents, antimetabolites, or recent radiotherapy.

**Table 1:** Baseline demographic and clinical characteristics of the four enrolled cohorts.

Cohort 1			
Characteristic	UCT(n=3)	NCTU(n=3)	P value
Age ,years (mean±SD)	60.33 ± 9.07	63.67 ± 9.29	0.679
Male sex, no (%)	2(66.67)	2(66.67)	1.000
Smoking status, no (%)	0(0)	0(0)	NA
Alcohol consumption, no (%)	0(0)	0(0)	NA
Comorbidity, no (%)	1(33.33)	1(33.33)	1.000
anti-TB treatment, no (%)	0(0)	0(0)	NA
Active tuberculosis, no (%)	0(0)	0(0)	NA
Impaired immune function, no (%)	0(0)	0(0)	NA
Immunosuppressive therapy, no (%)	0(0)	0(0)	NA
Abnormal liver function, no (%)	0(0)	0(0)	NA
Abnormal kidney function, no (%)	0(0)	0(0)	NA
Histopathological confirmation, no (%)	3(100)	3(100)	1.000
Cohort 2			
Characteristic	UCT(n=24)	NCTU(n=24)	P value
Age ,years (mean±SD)	49.42 ± 16.81	52.83 ± 22.27	0.552
Male sex, no (%)	9(37.50)	14(58.33)	0.149
Smoking status, no (%)	2(8.33)	3(12.5)	1.000
Alcohol consumption, no (%)	0(0)	0(0)	NA
Comorbidity, no (%)	10(41.67)	16(66.67)	0.083
anti-TB treatment, no (%)	0(0)	0(0)	NA
Active tuberculosis, no (%)	0(0)	0(0)	NA
Impaired immune function, no (%)	0(0)	0(0)	NA
Immunosuppressive therapy, no (%)	0(0)	0(0)	NA
Abnormal liver function, no (%)	0(0)	0(0)	NA
Abnormal kidney function, no (%)	2(8.33)	4(16.67)	0.663
Histopathological confirmation, no (%)	24(100)	24(100)	1.00
Cohort 3			
Characteristic	UCT(n=14)	NCTU(n=14)	P value
Age ,years (mean±SD)	46.29 ± 13.79	56.36 ± 19.39	0.125
Male sex, no (%)	5(35.71)	7(50.00)	0.704
Smoking status, no (%)	1(7.14)	1(7.14)	1.000
Alcohol consumption, no (%)	1(7.14)	2(14.29)	1.000
Comorbidity, no (%)	4(28.57)	7(50.00)	0.440
anti-TB treatment, no (%)	0(0)	0(0)	NA
Active tuberculosis, no (%)	0(0)	0(0)	NA
Impaired immune function, no (%)	0(0)	0(0)	NA
Immunosuppressive therapy, no (%)	0(0)	0(0)	NA
Abnormal liver function, no (%)	2(14.29)	0(0)	0.481
Abnormal kidney function, no (%)	1(7.14)	3(21.43)	0.596
Histopathological confirmation, no (%)	14(100)	14(100)	1.000
Cohort 4			
Characteristic	UCT(n=41)		
Age ,years (mean±SD)	40.41 ± 12.78		
Male sex, no (%)	15(36.59)		
Smoking status, no (%)	1(2.44)		
Alcohol consumption, no (%)	0(0)		
Comorbidity, no (%)	5(12.20)		
anti-TB treatment, no (%)	0(0)		
Active tuberculosis, no (%)	0(0)		
Impaired immune function, no (%)	0(0)		
Immunosuppressive therapy, no (%)	0(0)		
Abnormal liver function, no (%)	2(4.88)		
Abnormal kidney function, no (%)	0(0)		
Histopathological confirmation, no (%)	41(100)		

Abnormal liver function: ALT or AST > 2×ULN, or total bilirubin > 1.5×ULN; Abnormal kidney function: serum creatinine > 1.5 mg/dL (133 µmol/L) or eGFR < 60 mL/min/1.73 m²; UCT, ulcerative cutaneous tuberculosis; NCTU, non-tuberculous cutaneous ulcer; SD, standard deviation; TB, tuberculosis. No significant differences were observed between UCT and NCTU groups within each cohort (*P* > 0.05 for all comparisons).

### 2.2 Bioinformatics analysis of lncRNA *HCP*5 and mRNAs

#### 2.2.1 Microarray analysis

Tissue specimens from patients with UCT (n=3) and NCTU (n=3) were subjected to microarray analysis. Total RNA quantification was performed using a NanoDrop 2000 spectrophotometer (Thermo Scientific, USA), while RNA integrity was assessed via the Agilent 2100 Bioanalyzer (Agilent Technologies, USA). Sample labeling, hybridization, and washing strictly followed the manufacturer’s protocols for the Human LncRNA V6 Microarray (4×180K; design ID: 084410; Agilent Technologies, USA). Briefly, total RNA was reverse-transcribed into double-stranded cDNA, followed by *in vitro* transcription to synthesize cyanine-3-CTP-labeled complementary RNA (cRNA). The labeled cRNAs were then hybridized to the microarrays. Following the wash steps, arrays were scanned using the Agilent G2505C Microarray Scanner. Raw data extraction was conducted using Feature Extraction software v10.7.1.1, and subsequent data analysis was performed with GeneSpring v13.1 (Agilent Technologies, USA). Differentially expressed transcripts were defined as those with a fold change (FC) ≥ 2.0 and an unadjusted p−value ≤ 0.05.

#### 2.2.2 Functional enrichment analysis

To elucidate the biological roles of lncRNA HCP5, we conducted functional enrichment analyses on its co-expressed mRNAs. This encompassed Gene Ontology (GO, http://geneontology.org/) annotations—spanning Biological Process (BP), Cellular Component (CC), and Molecular Function (MF) categories—and Kyoto Encyclopedia of Genes and Genomes (KEGG, https://www.kegg.jp/) pathway analysis. Statistical significance was determined using the hypergeometric distribution test with a threshold of p < 0.05.

#### 2.2.3 ceRNA network construction

Functional enrichment analysis indicated that lncRNA HCP5 is significantly implicated in pathways associated with tuberculosis and immune system processes. To elucidate the underlying molecular mechanisms, we constructed a competing endogenous RNA (ceRNA) network centered on these specific pathways. Initially, mRNAs were selected based on their involvement in these pathways, differential expression (FC ≥ 5.0, p < 0.01), and significant co−expression with lncRNA HCP5 (|Pearson’s r| ≥ 0.8, p < 0.01). Potential miRNA response elements shared between lncRNA HCP5 and the selected mRNAs were then predicted using miRanda v3.3a (score ≥ 150, energy ≤ −30 kcal/mol). Finally, the ceRNA network was visualized using Cytoscape v3.9.1.

### 2.3 Clinical sample analysis and validation

#### 2.3.1 Real-time reverse transcription-quantitative polymerase chain reaction for tissue samples

Total RNA was extracted from the samples utilizing TRIzol^®^ Reagent (Thermo Fisher Scientific, USA). The concentration and purity of the RNA were evaluated through spectrophotometric analysis. For complementary DNA (cDNA) synthesis, 50 ng of total RNA was reverse-transcribed employing the HiScript™ II QRT SuperMix kit (Vazyme Biotech, China). Subsequently, quantitative real-time PCR (qPCR) was conducted on the synthesized cDNA using an iCycleriQ™ Real-Time PCR Detection System (Bio-Rad Laboratories, USA) under standard three-step thermal cycling conditions.

#### 2.3.2 Real-time RT-qPCR for plasma samples

Blood samples (2 mL) were collected in EDTA-coated tubes and processed within two hours post-collection. Plasma was separated by centrifugation at 2500 rpm for 5 minutes at 4 °C. The plasma supernatant was carefully aspirated, aliquoted into 1.5 mL microcentrifuge tubes, and stored at 4 °C for short-term preservation. Total RNA was then extracted from the plasma aliquots for reverse transcription quantitative PCR (RT-qPCR) analysis. Specifically, RNA was isolated using TRIzol LS Reagent (Thermo Fisher Scientific, USA) following the manufacturer’s protocol and quantified using a NanoDrop 2000 spectrophotometer (Thermo Fisher Scientific). Complementary DNA (cDNA) was synthesized from 50 ng of total RNA utilizing a reverse transcription kit (specify kit name and manufacturer), and subsequently stored at–20 °C until further analysis. Quantitative PCR (qPCR) was conducted on the synthesized cDNA employing the iCycler iQ™ Real-Time PCR Detection System (Bio-Rad Laboratories, USA), adhering to standard three-step thermal cycling parameters.

#### 2.3.3 Hematoxylin and eosin staining

Skin tissue samples harvested from the wounds were immediately placed on absorbent paper to prevent curling and fixed overnight in 10% neutral buffered formalin. Following fixation, the specimens were dehydrated through a graded ethanol series, embedded in paraffin, and sectioned at a thickness of 5 μm. The sections were deparaffinized in xylene and rehydrated through a descending ethanol gradient. After a 5-min rinse in distilled water, the slides were stained with hematoxylin for 3 min, followed by differentiation in ammonia water for 3 min to reduce nonspecific background staining. Subsequently, eosin staining was applied for 3 min, and a second ammonia water treatment was performed for 4 min. The sections were then dehydrated through increasing concentrations of ethanol (5 min each), cleared in xylene for 15 min, and coverslipped with neutral balsam. Stained sections were observed and imaged using a bright-field microscope (Nikon, Japan) at 200× magnification.

#### 2.3.4 Combined RNA *In situ* hybridization and immunofluorescence staining

RNA *in situ* hybridization (RNA-ISH) combined with immunofluorescence (IF) was performed to detect lncRNA *HCP5* expression and visualize cell nuclei in tissue sections. Tissue sections were deparaffinized, rehydrated, and treated with proteinase K for 10 min at room temperature. After PBS washing and ethanol dehydration, sections were hybridized with SweAMI HCP5 probe (Catalog No. GDP1087) diluted 1:50 in hybridization buffer. The probe mixture was denatured at 88 °C for 3 min followed by 37 °C for 5 min, then applied to sections, coverslipped, sealed, and incubated overnight at 37 °C. Post-hybridization, sections were washed sequentially with 2×, 1×, and 0.5× saline-sodium citrate buffer at 37 °C for 5 min each. For immunofluorescence, sections were blocked with 5% BSA for 30 min, incubated with primary antibody overnight at 4 °C, followed by fluorescent secondary antibody for 1 h at room temperature. Nuclei were counterstained with DAPI for 5 min. After mounting with anti-fade medium, images were captured using a fluorescence microscope (Zeiss, Germany).

### 2.4 Cellular validation of lncRNA HCP5 and downstream mRNAs

#### 2.4.1 Cell lines and cell culture

THP-1 cells were purchased from the China Center for Type Culture Collection (CCTCC) and cultured in 1640 medium + 10% fetal bovine Plasma at 37 °C with 5% CO_2_.The Mycobacterium tuberculosis vaccine strain BCG was obtained from Jingnuo Biological Company.

#### 2.4.2 THP-1 cell culture

RPMI-1640 medium, pre-warmed to 37 °C, was dispensed into a 15 mL conical centrifuge tube. A cryovial containing THP-1 monocytes was rapidly thawed by transferring it from liquid nitrogen storage into a 37 °C water bath, with gentle agitation until a small ice crystal remained. The vial was then removed, and its exterior surface was sterilized with 70% ethanol. The cell suspension was aseptically transferred to the prepared centrifuge tube containing the medium. The contents of the tube were gently mixed by pipetting and subsequently centrifuged at 800 rpm for 5 minutes at room temperature. Following centrifugation, the supernatant was carefully aspirated and discarded. The cell pellet was resuspended in 10 mL of fresh, pre-warmed complete medium. The cell suspension was thoroughly mixed and transferred to a 100 mm cell culture dish. The dish was then placed in a humidified incubator maintained at 37 °C with 5% CO_2_for culture.

#### 2.4.3 Establishment of a BCG-Infected macrophage model

THP-1 cells were harvested and subjected to centrifugation at 1000 rpm for 5 minutes to form a pellet. The resultant cell pellet was resuspended in complete medium and adjusted to a concentration of 2.5 × 10^5^ cells/mL. To induce differentiation, Phorbol 12-myristate 13-acetate (PMA) was introduced into the cell suspension at a final concentration of 5 ng/mL. Subsequently, the cells were distributed into a 24-well plate at a volume of 500 μL per well, corresponding to 1.25 × 10^5^ cells per well. The plate was incubated at 37 °C in a humidified atmosphere containing 5% CO_2_ for 24 hours to facilitate macrophage differentiation. Post-incubation, the supernatant containing PMA was carefully aspirated. The adherent macrophage monolayer was gently washed twice with Dulbecco’s Phosphate-Buffered Saline (DPBS) to eliminate residual PMA and non-adherent cells. Fresh complete growth medium devoid of PMA was subsequently added. The cells were then returned to the incubator (37 °C, 5% CO_2_) for a 72-hour resting period to ensure full differentiation into a quiescent macrophage state. Prior to infection, the medium was aspirated, and the cells were washed twice with DPBS. The infection process was initiated by adding fresh medium containing Mycobacterium bovis Bacillus Calmette–Guérin (BCG) at a multiplicity of infection (MOI) of 10. The infection was allowed to proceed by incubation under standard conditions (37 °C, 5% CO_2_).

#### 2.4.4 The expression level of lncRNA HCP5 in the BCG interfered macrophage model was detected by RT-qPCR

Macrophages were collected at predetermined intervals (0, 1, 4, 8, 12, 24, 48, and 72 hours) following infection with BCG. The culture supernatant was removed, and the cell monolayer was rinsed twice with ice-cold phosphate-buffered saline (PBS). Subsequently, total RNA was extracted using the TRIzol reagent, reverse-transcribed into complementary DNA (cDNA), and analyzed via quantitative reverse transcription PCR (RT-qPCR). The RT-qPCR assays were conducted utilizing the ChamQ™ SYBR^®^ qPCR Master Mix (Without ROX) (Vazyme, China) in accordance with the manufacturer’s instructions. The relative expression level of lncRNA *HCP5* was determined using the 2−ΔΔCT method, with glyceraldehyde-3-phosphate dehydrogenase (GAPDH) employed as the endogenous control.

#### 2.4.5 Plasmid construction

Short hairpin RNAs (shRNAs) targeting the human HLA complex P5 (HCP5; NR_040666.1) were designed using in silico methods. The most effective shRNA sequences were selected for synthesis using the siDirect version 2.0 algorithm. The specific shRNA sequences utilized in this study are detailed in **Table 1**. The lentiviral vector pLVX-U6-shRNA-mCherry-PuroR was digested with BamHI and XhoI restriction enzymes to produce linearized backbone fragments. The annealed oligonucleotides, corresponding to the shRNA sequences detailed in **Table 1**, underwent phosphorylation and were subsequently ligated into the digested vector backbone utilizing T4 DNA ligase. The resultant ligation product was transformed into DH5α competent *E. coli* cells. These transformed bacteria were plated on LB agar supplemented with 100 µg/mL ampicillin and incubated overnight. Individual colonies were then isolated, and plasmid DNA was extracted for sequence verification of positive clones.

#### 2.4.6 Lentivirus package

HEK293T cells were seeded at a density of 3 × 10^5^ cells/mL in a 100 mm cell culture dish and incubated at 37 °C for 24 hours to achieve approximately 70–80% confluency. Transfection was performed using polyethylenimine (PEI) at a PEI: DNA mass ratio of 4:1, in accordance with the manufacturer’s instructions. Six hours post-transfection, the medium was replaced with fresh complete medium. The lentivirus-containing supernatant was collected 72 hours later, clarified by filtration through a 0.45 μm pore-size membrane, and stored at 4 °C. For concentration, the clarified supernatant was combined with Lenti-X Concentrator (Takara Bio) at a 4:1 (supernatant:concentrator) volume ratio, mixed gently, and incubated at 4 °C for a period ranging from 2 hours to overnight. During the initial incubation phase, the mixture was gently inverted every 30 minutes for the first two hours. Subsequently, the mixture underwent centrifugation at 4000 rpm for 25 minutes at 4 °C. The supernatant was meticulously removed, and the lentiviral pellet was resuspended in a small volume (ranging from 1/50 to 1/100 of the original supernatant volume) of DMEM or ice-cold PBS. The pellet was gently resuspended via pipetting to ensure uniformity. The titer of the concentrated lentivirus was quantified, for instance, using quantitative PCR (qPCR) with lentiviral-specific primers. Aliquots of 50 µL were stored at –80 °C for long-term preservation.

#### 2.4.7 Screening of THP-1 stably transfected cells

THP-1 cells were seeded in 24-well plates at a density of 2×10^5^ cells/mL, with a multiplicity of infection (MOI) of 80 lentivirus-infected THP-1 cells. After a 24-hour incubation period, the medium was replaced, and incubation continued for an additional 72 hours, followed by the initiation of resistance screening. After 2 to 4 weeks of resistance screening, quantitative reverse transcription PCR (RT-qPCR) was employed to assess gene transcription levels.

#### 2.4.8 Infection of THP-1 cells with BCG

THP-1 cells, which had been transduced with BCG-shNC, BCG-shHCP5-1, or a non-targeting control (hereafter referred to as BCG, BCG-shNC, and BCG-shHCP5-1 cells), were seeded at a density of 2.5 × 10^5^ cells/mL in 100 mm culture dishes. Differentiation of these cells was induced by the addition of phorbol 12-myristate 13-acetate (PMA) to achieve a final concentration of 5 ng/mL, followed by incubation for 24 hours at 37°C in a humidified atmosphere containing 5% CO_2_. Post-differentiation, the PMA-containing medium was carefully aspirated, and the resulting adherent macrophage monolayer was gently washed twice with Dulbecco’s Hanks’ Balanced Salt Solution (D-HBSS) to eliminate residual inducer and non-adherent cells. The macrophages were subsequently infected by replacing the medium with RPMI-1640 medium supplemented with 10% fetal bovine Plasma (FBS) containing Mycobacterium bovis BCG at a multiplicity of infection (MOI) of 10. The infection was permitted to proceed for 2 hours under standard culture conditions (37 °C, 5% CO_2_). Thereafter, extracellular bacteria were removed by aspirating the supernatant, washing the cells twice with D-HBSS, and introducing fresh medium containing 30 µg/mL gentamicin to eradicate any remaining extracellular BCG. After a 24-hour incubation in gentamicin-containing medium, cells were harvested for RNA extraction and subsequent RT-qPCR analysis.

#### 2.4.9 Real-time reverse transcription-quantitative polymerase chain reaction RT-qPCR for cell samples

THP-1-shHCP5 macrophages were collected 24 hours following infection with BCG. The culture supernatant was removed, and the cells underwent two washes with ice-cold phosphate-buffered saline (PBS). Total RNA was subsequently extracted using TRIzol reagent and reverse-transcribed into complementary DNA (cDNA). Quantitative reverse transcription PCR (RT-qPCR) was conducted utilizing the ChamQ™ SYBR^®^ qPCR Master Mix (Without ROX) (Vazyme, China), employing the synthesized cDNA as the template. The relative mRNA expression levels of MYD88, ITGB2, CORO1A, and HLA-DRB1 were quantified using the 2−ΔΔCT method, with glyceraldehyde-3-phosphate dehydrogenase (GAPDH) serving as the endogenous control.

### 2.5 Clinical sample validation during treatment

#### 2.5.1 Real-time reverse transcription-quantitative polymerase chain reaction for tissue and plasma samples

The expression levels of lncRNA *HCP5* and *ITGB2* in the tissues and Plasma of 41 UCT patients before and after treatment were detected and analyzed by RT-qPCR.

#### 2.5.2 Immunohistochemical analysis

The protein expression of ITGB2 was assessed through immunohistochemistry (IHC) in lesional skin biopsies obtained from 24 UCT patients, both prior to and following a 2-week regimen of anti-tuberculosis therapy. Formalin-fixed, paraffin-embedded (FFPE) tissue blocks were sectioned at a thickness of 4 μm. The sections were subsequently deparaffinized in xylene and rehydrated through a graded ethanol series. Antigen retrieval was achieved by heating the sections in a citrate-based antigen retrieval buffer (pH 6.0; Servicebio, Wuhan, China). Endogenous peroxidase activity was inhibited by incubating the sections with 3% hydrogen peroxide. To prevent non-specific binding, the sections were subsequently incubated with 3% bovine Plasma albumin (BSA) in phosphate-buffered saline (PBS) for 30 minutes at room temperature. The sections were then incubated overnight at 4 °C with a primary antibody targeting ITGB2, with details regarding the catalog number, species, and dilution to be provided, diluted in PBS. This step was followed by a 50-minute incubation at room temperature with a horseradish peroxidase (HRP)-conjugated secondary antibody, diluted at 1:200, which was raised against the host species of the primary antibody. Immunoreactivity was detected using 3,3’-diaminobenzidine (DAB; Servicebio) as the chromogen, and the sections were counterstained with hematoxylin. Subsequently, the sections underwent dehydration, clearing in xylene, mounting with a neutral resinous medium, and were scanned using a digital slide scanner.

### 2.6 Statistical analyses

Statistical analyses were conducted using SPSS version 23.0 (IBM, Armonk, NY), employing Student’s t-test, one-way analysis of variance, and chi-squared test for intergroup comparisons. A p-value of less than 0.05 was considered indicative of statistical significance.

## 3 Results

### 3.1 The differences in the overall genome between the UCT and NCTU tissues

**Figure 2:**
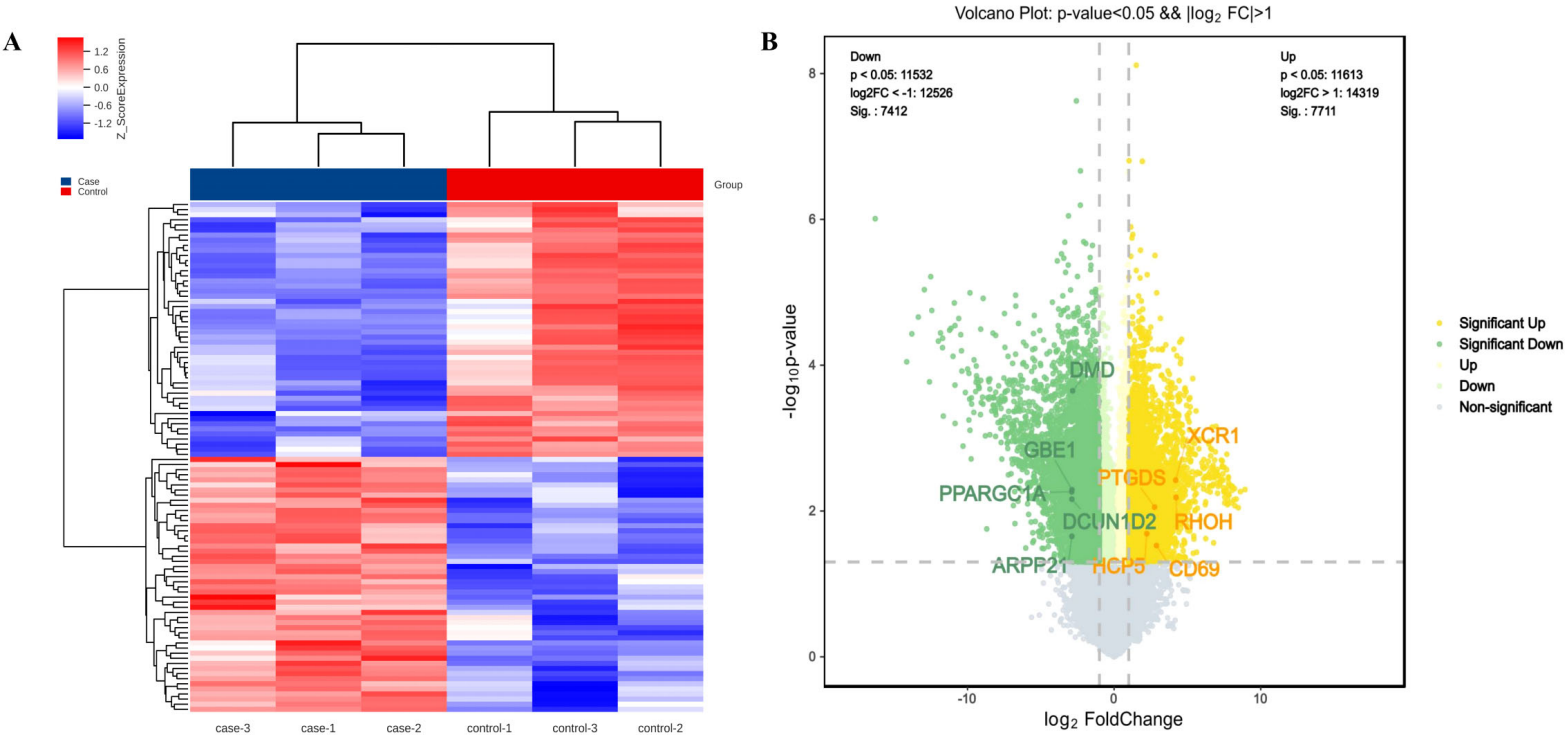
Identification and visualization of differentially expressed lncRNAs in UCT versus NCTU tissues. **(A)** Hierarchical clustering heatmap illustrating the expression patterns of the top 50 upregulated and top 50 downregulated lncRNAs (criteria: |log_2_Fold Change| > 1, *P* < 0.05). The color scale ranges from blue (low expression) to red (high expression). **(B)** Volcano plot depicting the global distribution of transcriptomic changes. Green and yellow dots represent significantly downregulated and upregulated lncRNAs, respectively. The top five most significant lncRNAs in each direction are explicitly annotated. FC, fold change; UCT, ulcerative cutaneous tuberculosis; NCTU, non-tuberculous cutaneous ulcer.

To elucidate the regulatory mechanisms of long non-coding RNAs (lncRNAs) in UCT, a comprehensive transcriptomic analysis was performed, comparing the expression profiles of lncRNAs between UCT and NCTU tissues. This analysis revealed 1,994 differentially expressed lncRNA transcripts, with 1,290 exhibiting upregulation and 704 demonstrating downregulation (|log_2_FC| > 1, *p* < 0.05). To concentrate on the most significantly altered transcripts, the top 50 upregulated and top 50 downregulated lncRNAs, ranked by absolute fold change, were selected for further visualization. Their expression patterns were depicted using a volcano plot and a hierarchical clustering heatmap (**Figure 2**). In the volcano plot, the five most upregulated and five most downregulated lncRNAs from this subset were specifically annotated to underscore their potential biological significance.

Among these differentially expressed transcripts, lncRNA HCP5 emerged as one of the most significantly upregulated candidates in our sequencing data (**Figure 2**). Notably, lncRNA HCP5 is situated within the Major Histocompatibility Complex Class I region, which plays a pivotal role in the host immune response against Mycobacterium tuberculosis. Furthermore, the specific functional role of lncRNA HCP5 in UCT remains largely unexplored, representing a critical gap in the current literature. Consequently, lncRNA HCP5 was prioritized for subsequent functional investigation.

**Figure 3:**
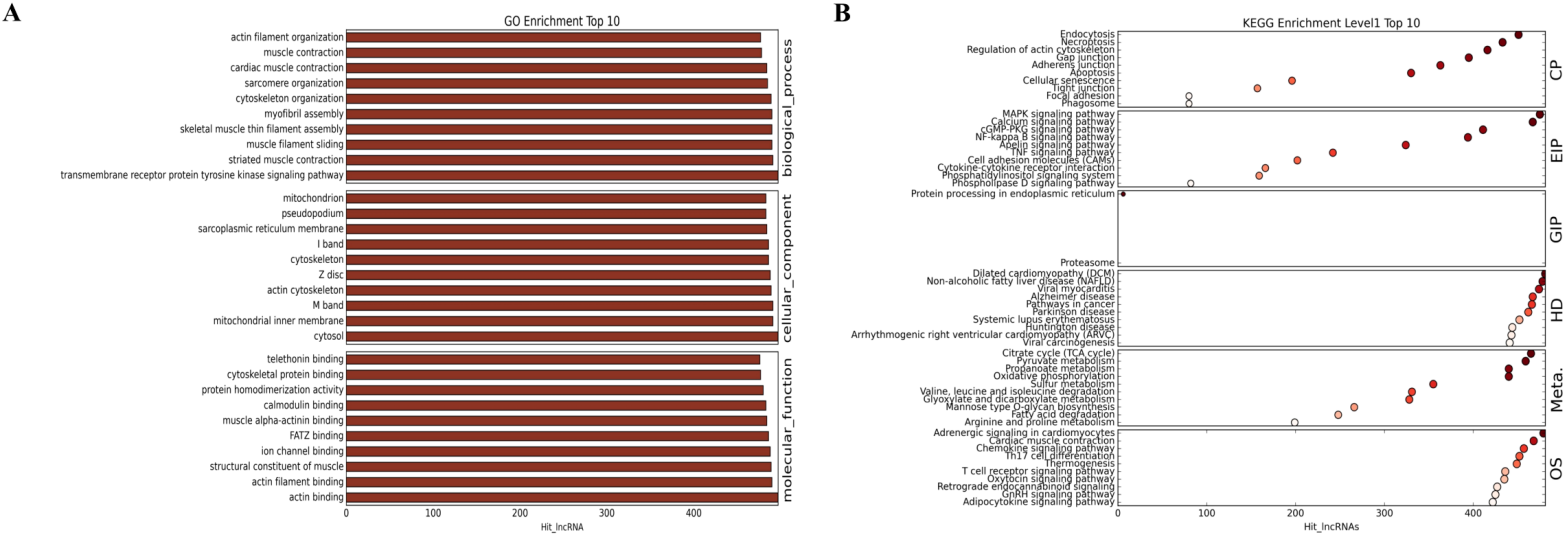
Functional enrichment analysis of lncRNA HCP5 co-expressed genes. **(A)** GO bar chart showing the top 10 enriched terms in Biological Process (BP), Cellular Component (CC), and Molecular Function (MF). Bar height is inversely proportional to p−value. **(B)** KEGG pathway bubble chart. Dot size represents the number of genes enriched in each pathway; color indicates enrichment significance (red: smaller p−value). Pathways related to immune regulation, inflammation, and tuberculosis are highlighted. Statistical significance was set at *P* < 0.05. GO, Gene Ontology; KEGG, Kyoto Encyclopedia of Genes and Genomes.

The biological functions and signaling pathways linked to mRNAs co-expressed with the candidate long non-coding RNAs (lncRNAs) were explored using Gene Ontology (GO) and Kyoto Encyclopedia of Genes and Genomes (KEGG) enrichment analyses. The GO enrichment analysis for mRNAs associated with lncRNA HCP5 identified significant terms across three categories. In the Biological Processes (BP) category, terms related to cytoskeleton organization and actin filament organization were enriched. The Cellular Component (CC) category showed enrichment for the cytosol, cytoskeleton, pseudopodium, and mitochondrion. The Molecular Function (MF) category was significantly enriched for actin binding, actin filament binding, and cytoskeletal protein binding (**Figure 3A**). The KEGG pathway analysis identified numerous significantly enriched pathways (p ≤ 0.05), which were subsequently categorized into the primary KEGG classifications. Notably, a significant subset of these pathways was associated with immune regulation, inflammatory signaling, and tissue repair processes. Key enriched pathways within this subset included those related to immune cell function (e.g., Phagosome, Cell adhesion molecules), inflammatory signaling (e.g., NF-κB signaling pathway, MAPK signaling pathway), and immune-repair coordination (e.g., T cell receptor signaling pathway, Chemokine signaling pathway) (**Figure 3B**).

**Figure 4:**
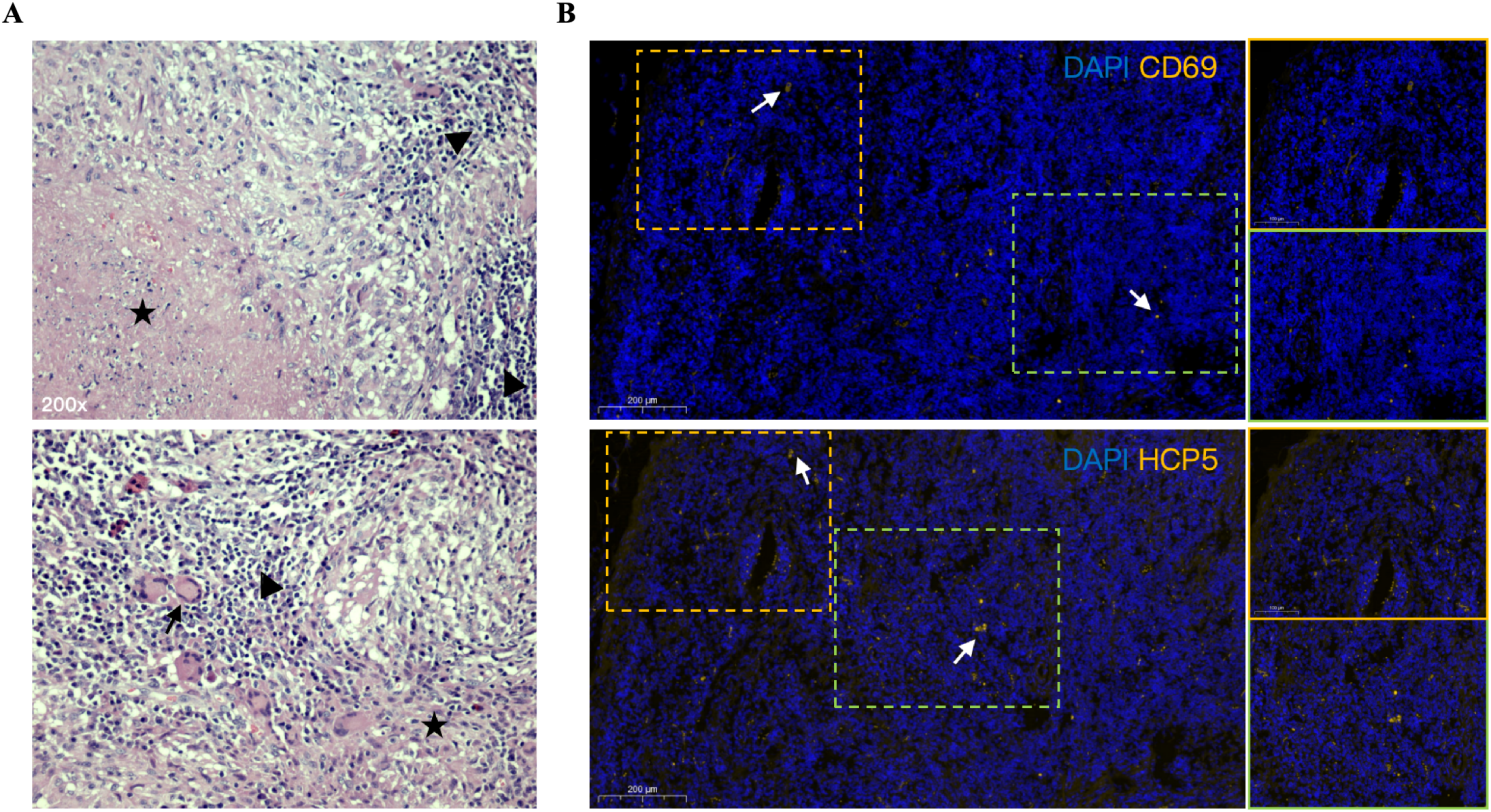
H&E staining and fluorescence *in situ* hybridization combined with immunofluorescence of UCT tissues. **(A)** H&E staining of UCT tissues (200×). Asterisk indicate necrotic areas; arrowheads mark dense cellular infiltration with deeply stained pleomorphic nuclei. **(B)** Spatial distribution of lnc RNA *HCP5* and *CD69* in UCT tissues detected by fluorescence *in situ* hybridization combined with immunofluorescence. Yellow: *CD69* (up), lncRNA *HCP5* (down); Blue: DAPI (nuclei). Arrows indicate CD69/lncRNA HCP5-positive cells. The merged image shows differential expression patterns, with lncRNA *HCP5* being more abundant than CD69 in the same lesion. Scale bar: 200 μm. UCT, ulcerative cutaneous tuberculosis.

H&E staining of tissue sections from two patients clinically diagnosed with cutaneous tuberculous ulcers revealed dense cellular infiltration with deeply stained nuclei and pleomorphic morphology, accompanied by necrotic areas and fibrous tissue, consistent with the pathological features of tuberculous lymphadenitis (**Figure 4A**). To further investigate the molecular signatures underlying tuberculous ulcer pathogenesis, we performed immunofluorescence combined with FISH to detect gene sequences significantly expressed in the lesional tissues. Notably, in the same patient lesions, the lncRNA *HCP5* exhibited markedly higher expression compared to *CD69*, suggesting that lncRNA *HCP5* may play a more prominent role than *CD69* in the progression of cutaneous tuberculous ulcers (**Figure 4B**). This differential expression pattern implies a potential regulatory function for lncRNA *HCP5* in the host immune response to mycobacterial infection, warranting further investigation into its mechanistic involvement in tuberculosis pathogenesis.

### 3.2 Establishment of lncHCP5-miRNA-mRNA network

**Figure 5:**
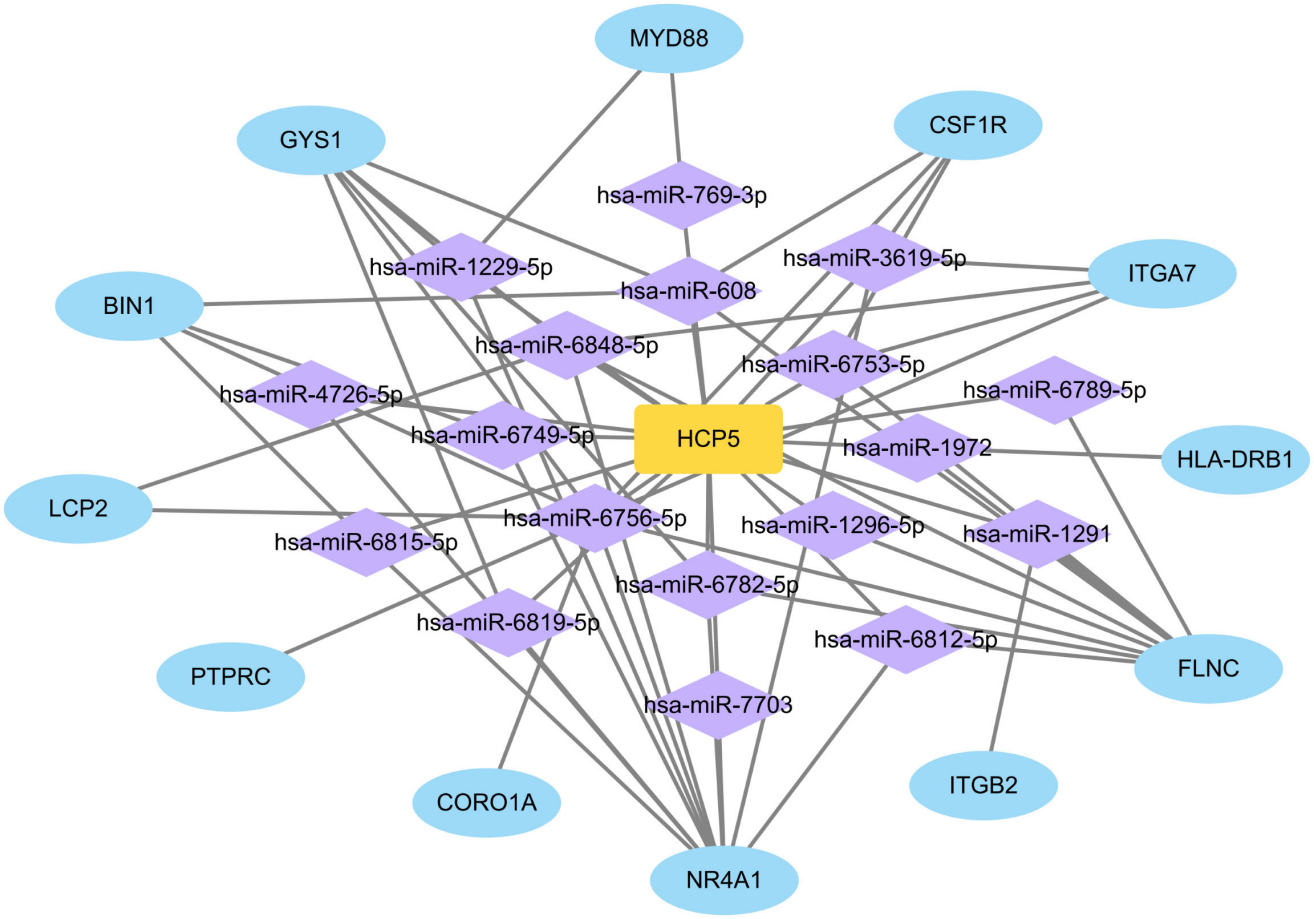
Construction of the lncRNA HCP5-miRNA-mRNA co-expression network. The network was constructed based on the correlation analysis (Pearson’s correlation coefficient > 0.8, *P* < 0.05). Red nodes represent lncRNA HCP5, yellow nodes represent miRNAs, and blue nodes represent target mRNAs. The thickness of the lines indicates the strength of the correlation. The network was visualized using Cytoscape v3.9.1.

Through transcriptomic analysis, it was determined that the genes differentially expressed in UCT, as opposed to NCTU, were significantly enriched in biological processes associated with immune response, inflammation, and wound healing. To explore potential regulatory mechanisms, a co-expression network was constructed to associate lncRNA *HCP5* with putative mRNA targets involved in these processes. By applying stringent correlation criteria, we identified 12 downstream mRNAs exhibiting strong co-expression with lncRNA *HCP5* (**Figure 5**).

### 3.3 Differential expression of lncRNA *HCP5* in wound lesion tissues and plasma between patients with ulcerative cutaneous tuberculosis and those with non-tuberculous cutaneous ulcers

**Figure 6:**
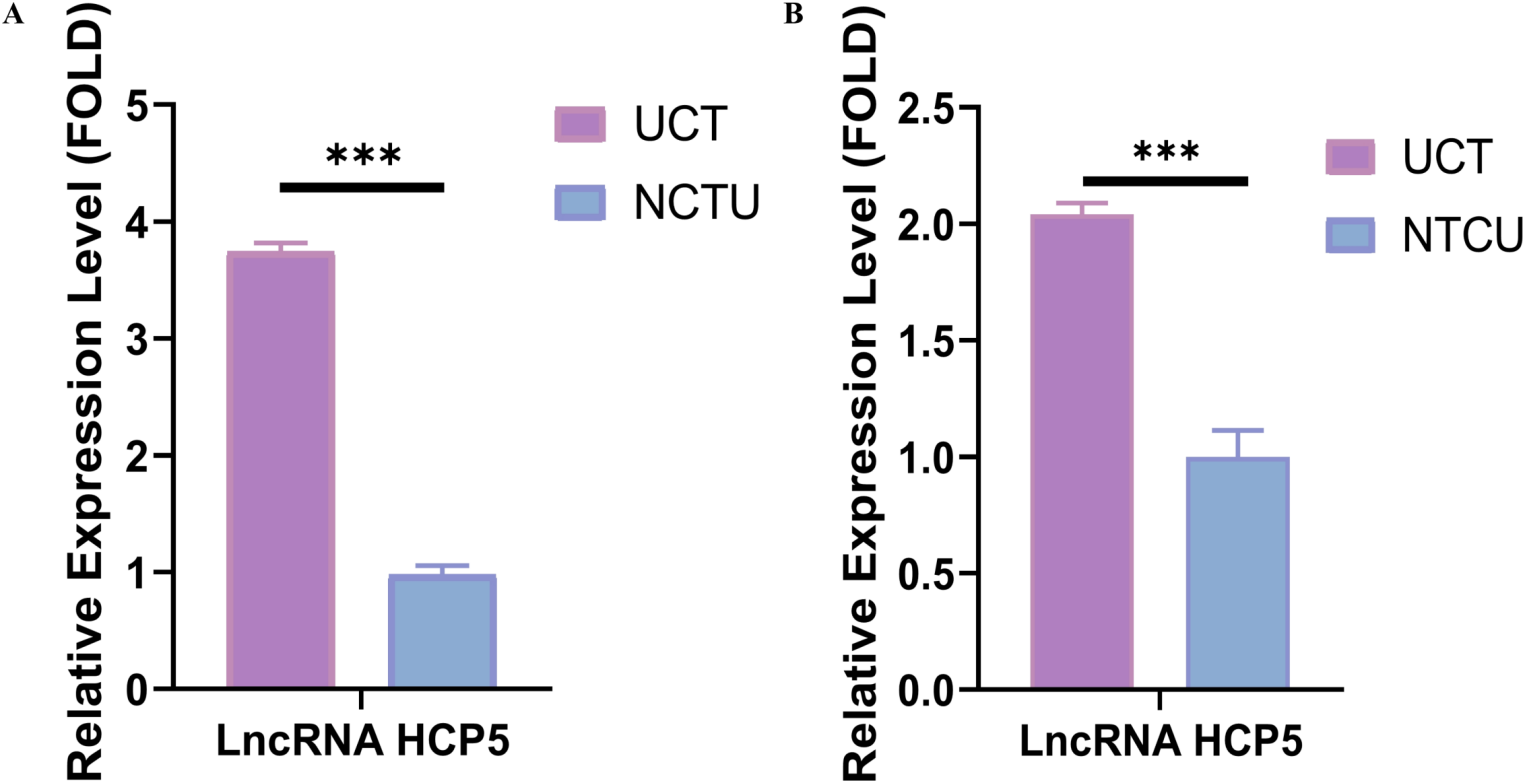
Differential expression of lncRNA *HCP5* in tissue and plasma cohorts. **(A)** Relative expression levels of lncRNA *HCP5* in cutaneous lesion tissues from patients with Ulcerative Cutaneous Tuberculosis (UCT, n=24) and Non-Tuberculous Cutaneous Ulcers (NCTU, n=24). **(B)** Relative expression levels of lncRNA *HCP5* in plasma specimens from the same cohorts. Data are presented as mean ± SEM. The ratio of the expression level of the two groups (*P* < 0.01). UCT, ulcerative cutaneous tuberculosis; NCTU, non-tuberculous cutaneous ulcer. ***P < 0.001.

To validate the transcriptomic findings, we quantified lncRNA *HCP5* expression in an independent cohort (Cohort 2, n=24 per group). As shown in **Figure 6A**, lncRNA *HCP5* was significantly upregulated in UCT tissues compared to NCTU controls, with a 3.74-fold increase (3.74 ± 0.08 vs. 1.00 ± 0.08, *P* < 0.001; **Table 2**). Consistent with this tissue-level elevation, plasma lncRNA *HCP5* levels were also markedly higher in UCT patients (**Figure 6B**), showing a 2.04-fold increase (2.04 ± 0.05 vs. 1.00 ± 0.11, *P* < 0.001; **Table 2**). The concordant upregulation of lncRNA *HCP5* in both tissue and plasma, together with the highly significant P values, strongly supports the potential of circulating lncRNA *HCP5* as a non-invasive diagnostic biomarker for UCT. Furthermore, the magnitude of upregulation (nearly 4-fold in tissue and 2-fold in plasma) suggests that lncRNA HCP5 is not merely a passive marker but may be functionally involved in the disease process.

**Table 2:** Expression profiles of lncRNA HCP5-correlated target mRNAs in clinical tissues from Cohorts 2 and 3.

Biomarker	Sample type	n	UCT (mean±SEM)	NCTU (mean±SEM)	P-value
lncRNA HCP5	Tissue (relative expression, 2^(-ΔΔCt))	24	3.74 ± 0.08	1.00 ± 0.08	<0.001
lncRNA HCP5	Plasma (relative expression)	24	2.04 ± 0.05	1.00 ± 0.11	<0.001
MYD88 mRNA	Tissue (relative expression)	14	0.68 ± 0.17	1.00 ± 0.33	0.394
HLA-DRB1 mRNA	Tissue (relative expression)	14	3.92 ± 0.77	1.00 ± 0.25	0.001
ITGB2 mRNA	Tissue (relative expression)	14	3.93 ± 0.82	1.00 ± 0.40	0.004
CORO1A mRNA	Tissue (relative expression)	14	1.54 ± 0.30	1.00 ± 0.26	0.186

Data are presented as mean ± SEM. Relative expression was calculated using the 2^(-ΔΔCt) method with GAPDH as internal control and NCTU group as calibrator (set to 1.00). For lncRNA HCP5 (tissue and plasma), n=24 per group (Cohort 2); for MYD88, HLA-DRB1, ITGB2, and CORO1A, n=14 per group (Cohort 3). *P* < 0.05 was considered statistically significant. UCT, ulcerative cutaneous tuberculosis; NCTU, non−tuberculous cutaneous ulcer; SEM, standard error of the mean.

### 3.4 Correlation of lncRNA *HCP5* and its potential target mRNAs

**Figure 7:**
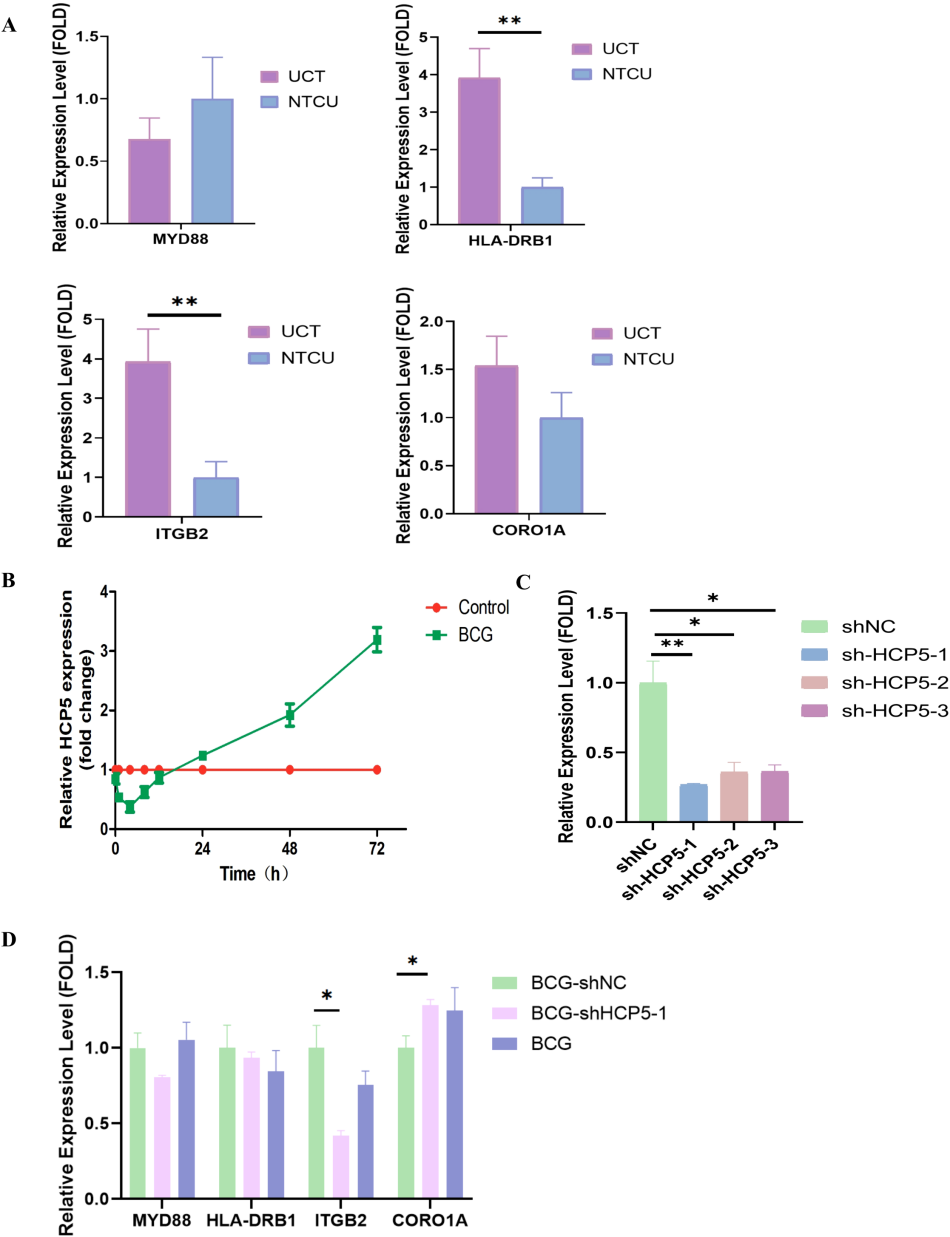
Correlation of lncRNA *HCP5* with predicted target mRNAs and cellular validation. **(A)** Relative expression of *MYD88*, *HLA-DRB1*, *ITGB2*, and *CORO1A* in UCT (n=14) vs. NCTU (n=14) tissues (Cohort 3). Data are presented as mean ± SEM. p<0.01 vs. NCTU. **(B)** Time-dependent expression of lncRNA *HCP5* in BCG-infected THP−1 macrophages (MOI = 10). Expression was normalized to 0 h (set to 1.00). Data are mean ± SEM (n=3). **(C)** Knockdown efficiency of three independent shRNAs targeting lncRNA *HCP5*. sh-HCP5-1 reduced HCP5 expression to 0.27 ± 0.01-fold of shNC control (*P* < 0.01). Data are mean ± SEM (n=3). **(D)** Effect of *HCP5* knockdown (sh-HCP5-1) on downstream target gene expression in BCG-infected macrophages. Expression levels were normalized to BCG-shNC (set to 1.00). Data are mean ± SEM (n=3). *P* < 0.05 vs. BCG-shNC.UCT, ulcerative cutaneous tuberculosis; NCTU, non-tuberculous cutaneous ulcer; BCG, Bacillus Calmette-Guérin; MOI, multiplicity of infection; shNC, non-targeting short hairpin RNA control; SEM, standard error of the mean. *P < 0.05, **P < 0.01.

Through co-expression network analysis, four mRNAs—*MYD88*, *HLA-DRB1*, *ITGB2*, and *CORO1A*—associated with immune and inflammatory regulation were identified for experimental validation. RNA sequencing data revealed a positive correlation between the expression levels of these genes and the expression of the lncRNA *HCP5*. Validation of these correlations was performed using quantitative reverse transcription PCR (RT-qPCR) on tissue samples from 14 patients with ulcerative colitis (UCT) and 14 non-colitis tissue ulcer (NCTU) controls. Consistent with the sequencing data, the mRNA levels of *HLA-DRB1* and *ITGB2* were significantly elevated in UCT tissues compared to NCTU controls (**Figure 7A**; **Table 3**). Although the expression pattern of *CORO1A* was in agreement with the sequencing data, it did not achieve statistical significance (**Figure 7A**; **Table 3**). Conversely, *MYD88* expression exhibited a trend that diverged from the sequencing data and was not statistically significant (**Figure 7A**; **Table 3**). These findings indicate that HLA-DRB1 and ITGB2 may serve as potential regulators of impaired healing in UCT.

**Table 3:** Effect of lncRNA HCP5 knockdown on downstream target gene expression in THP-1 macrophages.

Group / Time	Biomarker	n	Relative expression (mean±SEM)	P value
Knockdown efficiency				
shNC(control)	lncRNA HCP5	3	1.00 ± 0.16	NA
sh-HCP5-1	lncRNA HCP5	3	0.27 ± 0.01	0.009
sh-HCP5-2	lncRNA HCP5	3	0.36 ± 0.07	0.019
sh-HCP5-3	lncRNA HCP5	3	0.37 ± 0.05	0.017
LncRNA HCP5 knockdown on target genes				
BCG-shNC(control)	MYD88 mRNA	3	1.00 ± 0.09	NA
BCG-shHCP5-1	MYD88 mRNA	3	0.81 ± 0.01	0.125
BCG	MYD88 mRNA	3	1.05 ± 0.12	0.743
BCG-shNC(control)	HLA-DRB1 mRNA	3	1.00 ± 0.15	NA
BCG-shHCP5-1	HLA-DRB1 mRNA	3	0.93 ± 0.04	0.693
BCG	HLA-DRB1 mRNA	3	0.85 ± 0.14	0.491
BCG-shNC(control)	ITGB2 mRNA	3	1.00 ± 0.15	NA
BCG-shHCP5-1	ITGB2 mRNA	3	0.42 ± 0.03	0.019
BCG	ITGB2 mRNA	3	0.76 ± 0.09	0.235
BCG-shNC(control)	CORO1A mRNA	3	1.00 ± 0.08	NA
BCG-shHCP5-1	CORO1A mRNA	3	1.28 ± 0.04	0.034
BCG	CORO1A mRNA	3	1.25 ± 0.15	0.227

Data are mean ± SEM (n = 3). Relative expression was calculated using the 2^(-ΔΔCt) method with GAPDH as internal control. For knockdown efficiency, expression was normalized to the shNC group. For target gene expression, expression was normalized to the BCG-shNC group. *P* < 0.05 was considered statistically significant. BCG, Bacillus Calmette-Guérin; shNC, non−targeting short hairpin RNA control; SEM, standard error of the mean.

To investigate the dynamic regulation of lncRNA HCP5, a BCG-infected macrophage model derived from human THP-1 cells differentiated with phorbol 12-myristate 13-acetate (PMA) was established. The expression of lncRNA *HCP5* was observed to increase in a time-dependent manner following BCG infection (**Figure 7B**; **Table 3**). Considering the potential roles of MYD88, CORO1A, HLA-DRB1, and ITGB2 in macrophage function during BCG infection, we subsequently explored their relationship with lncRNA HCP5 through a loss-of-function approach. A knockdown plasmid targeting lncRNA HCP5 was constructed, and RT-qPCR analysis confirmed that sh-HCP5-1 exhibited the highest knockdown efficiency, reducing lncRNA HCP5 expression to 0.27 ± 0.01-fold of the shNC control (*P* < 0.01; **Figure 7C**; **Table 3**), making it the choice for subsequent functional analyses. A BCG-infected macrophage model was then generated using PMA-differentiated THP-1 cells transduced with sh-HCP5-1. Upon lncRNA *HCP5* knockdown, there was a significant upregulation of *CORO1A* expression (p < 0.05; **Figure 7D**; **Table 3**), which was inconsistent with the positive correlation observed in the sequencing data. Conversely, *ITGB2* expression was significantly downregulated (p < 0.05; **Figure 7D**; **Table 3**), consistent with the positive correlation from sequencing data and its upregulation in clinical UCT samples. The expression changes of *MYD88* and *HLA-DRB1* were directionally consistent with the sequencing correlation; however, these changes were not statistically significant (**Figure 7D**; **Table 3**).

### 3.5 Analysis of expression of lncRNA *HCP5* and *ITGB2* during treatment

**Figure 8:**
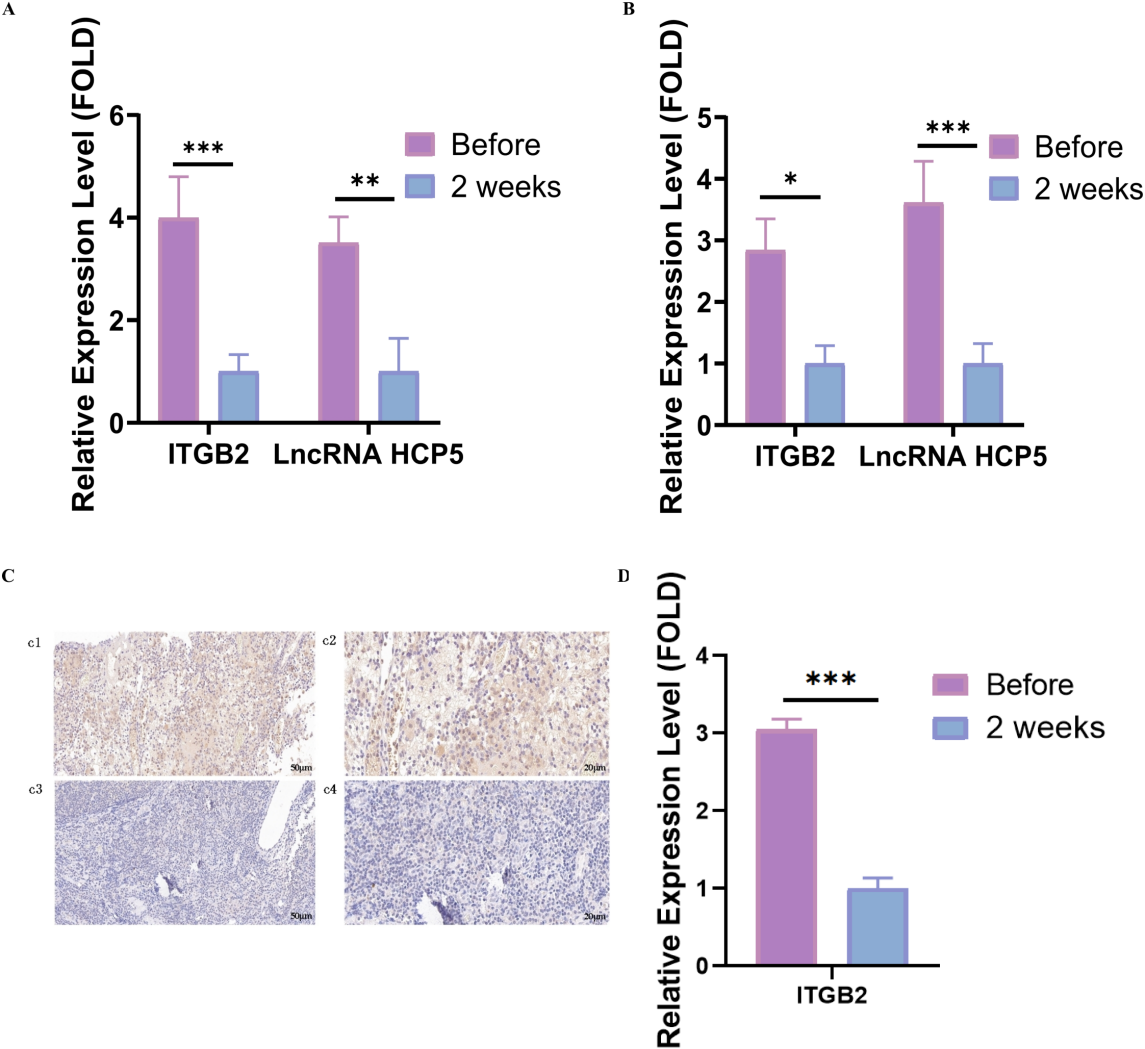
Dynamic changes of lncRNA HCP5 and ITGB2 during anti-tuberculosis treatment. **(A)** RT-qPCR analysis of lncRNA *HCP5* and *ITGB2* mRNA tissue samples before (Pre) and after 2 weeks of treatment (Post). (n = 41, *P* < 0.05 or *P* < 0.01). **(B)** RT-qPCR analysis of lncRNA *HCP5* and *ITGB2* mRNA plasma samples before (Pre) and after 2 weeks of treatment (Post). The ratio of the expression level of the two groups (n = 41, *P* < 0.05 or *P* < 0.01). **(C)** Representative immunohistochemistry (IHC) images of ITGB2 protein in UCT lesions pre-treatment (c1,c2) and post-treatment (c3,c4). Original magnifications: ×20 (c1,c3); ×40 (c2,c4). **(D)** Quantification of ITGB2 IHC positive area ratio (%). (P < 0.01).All data are presented as mean ± SEM. Pre, baseline before treatment; Post, 2 weeks after treatment initiation. *P < 0.05, **P < 0.01, ***P < 0.001.

To validate biomarker dynamics during therapy, we measured the expression of lncRNA *HCP5* and *ITGB2* by RT-qPCR in paired tissue and plasma samples from 41 UCT patients, collected before and after a 2-week course of anti-tuberculosis treatment. Following two weeks of treatment, tissue lncRNA *HCP5* expression significantly declined from 3.51 ± 0.51 to 1.00 ± 0.65 (3.51-fold; *P*<0.01; **Figure 8A**, **Table 4**). Similarly, plasma lncRNA *HCP5* levels dropped from 3.62 ± 0.67 to 1.00 ± 0.32 (3.62-fold; *P* < 0.01; **Figure 8B**; **Table 4**). ITGB2 followed a consistent pattern (**Figures 8A, B**). Tissue *ITGB2* mRNA decreased by 4.00-fold (P < 0.01), and plasma *ITGB2* mRNA decreased by 2.84-fold (*P*<0.01; **Table 4**). Furthermore, IHC analysis confirmed a significant reduction in ITGB2 protein expression (positive area: 3.05 ± 0.13 vs. 1.00 ± 0.14; *P* < 0.01; **Figures 8C, D**; **Table 4**). These data demonstrate that lncRNA HCP5 and ITGB2 are rapidly downregulated upon effective treatment, highlighting their utility as dynamic, non−invasive markers for monitoring therapeutic response in UCT.

**Table 4:** Longitudinal profiles of lncRNA HCP5 and ITGB2 expression in UCT patients (Cohort 4) during the first two weeks of anti-tuberculosis treatment.

Biomarker	Sample type	Baseline (Pre-treatment) (mean±SEM)	Week 2 (Post-treatment) (mean±SEM)	P-value
lncRNA HCP5	Tissue (relative expression, 2^(-ΔΔCt))	3.51±0.51	1.00±0.65	0.003
lncRNA HCP5	Plasma (relative expression)	3.62±0.67	1.00±0.32	<0.001
ITGB2 mRNA	Tissue (relative expression)	4.00±0.80	1.00±0.33	<0.001
ITGB2 mRNA	Plasma (relative expression)	2.84±0.51	1.00±0.29	0.002
ITGB2 protein	Tissue (relative expression, %Area ratio)	3.05±0.13	1.00±0.14	<0.001

Data are presented as mean±SEM. For lncRNA HCP5 and ITGB2: relative expression was calculated using the 2^(-ΔΔCt) method with GAPDH as internal control and normalized to the post−treatment time point (set to 1.00). For ITGB2 protein: expression was quantified by IHC as the percentage of positive area (brown staining) relative to total tissue area, and normalized to the post−treatment value (set to 1.00); n = 41 patients (Cohort 4). P < 0.05 was considered statistically significant. UCT, ulcerative cutaneous tuberculosis; SEM, standard error of the mean; IHC, immunohistochemistry.

## 4 Discussion

Our study provides the first comprehensive analysis of the long non-coding RNA *HCP5* in Ulcerative Cutaneous Tuberculosis (UCT), establishing its dual potential as a diagnostic biomarker and a monitor of therapeutic response. Through integrated bioinformatics analysis of clinical samples from UCT and Non-Tuberculous Cutaneous Ulcer (NTCU) patients, we identified a landscape of 1994 differentially expressed lncRNAs. Within this profile, lncRNA *HCP5* emerged as a prominently upregulated transcript, warranting focused investigation. This finding is particularly significant as lncRNA *HCP5*, encoded within the Major Histocompatibility Complex (MHC) class I region, has previously been linked to the host immune response against Mycobacterium tuberculosis, with evidence suggesting its expression facilitates bacterial survival within macrophages (Masoudian et al., 2015). Our discovery of its marked upregulation in UCT lesions directly extends this molecular link to the cutaneous form of the disease.

To demonstrate the functional role of lncRNA *HCP5*, we constructed a co-expression network that connected it to key biological processes dysregulated in UCT, namely immune response, inflammation, and impaired wound healing. This network pinpointed 12 strongly correlated downstream mRNAs. We strategically focused on four hub genes—*MYD88*, *HLA-DRB1*, *ITGB2*, and *CORO1A*—each with well-documented roles in pulmonary tuberculosis pathogenesis but unexplored in the context of UCT. Experimental validation in patient tissues confirmed the significant upregulation of *HLA-DRB1* and *ITGB2*, aligning with both our sequencing data and their known pro-inflammatory functions. Furthermore, using an *in vitro* BCG-infected macrophage model, we demonstrated that lncRNA *HCP5* expression is dynamically induced in a time-dependent manner upon infection. Crucially, functional loss-of-function studies revealed that lncRNA *HCP5* knockdown led to a significant decrease in ITGB2 expression. This coordinated regulation identifies a novel lncRNA *HCP5*/*ITGB2* regulatory axis, proposing a specific mechanism by which lncRNA *HCP5* may exacerbate local inflammation and impede tissue repair in UCT by modulating integrin-mediated leukocyte adhesion and phagocytic functions.

The translation of this molecular insight addresses a pressing clinical gap. Unlike pulmonary tuberculosis, the management of extrapulmonary TB (EPTB), including UCT, suffers from the absence of standardized, objective metrics for evaluating treatment response, contributing to high rates of recurrence and suboptimal outcomes (Klingmüller et al., 2025; Khalid et al., 2023; Sinha et al., 2025). Our longitudinal clinical analysis directly confronts this challenge. By serially profiling tissue and plasma samples from UCT patients undergoing therapy, we demonstrated that the expression levels of both lncRNA *HCP5* and its downstream target *ITGB2* decreased significantly after just two weeks of anti-tuberculosis treatment. This rapid, coordinated decline highlights their dynamic sensitivity to therapeutic intervention and underscores their exceptional promise as a novel, objective biomarker pair. In summary, this work not only uncovers a novel pathogenic axis in UCT but also pioneers the development of lncRNA *HCP5* and *ITGB2* as concrete tools for improving the diagnosis and personalized management of this challenging form of cutaneous tuberculosis.

## 5 Conclusion

In conclusion, this integrated investigation—combining bioinformatics discovery, clinical validation, and functional mechanistic studies—has established the lncRNA *HCP5*/*ITGB2* axis as a critical regulator in the pathogenesis of UCT. The rapid, treatment-responsive downregulation of both molecules underscores their strong potential as clinically actionable biomarkers for real-time monitoring of therapeutic efficacy. Thus, our findings advance the molecular understanding of UCT and provide a practical translational strategy to overcome current limitations in clinical management, ultimately offering a pathway to improve patient outcomes.

## Data Availability

The datasets analyzed during the current study are available in the NCBI Gene Expression Omnibus (GEO) repository, under the accession number GSE331511.
